# Racial disparities in time to treatment initiation and survival outcomes in patients with vulvar cancer: a national cancer database analysis

**DOI:** 10.1007/s10147-026-03111-1

**Published:** 2026-07-06

**Authors:** Abdelrahman Yousif, Hatem S. Mohamed, Mohanad Elchouemi, Sanjana Reddy Mada, Ilana Chefetz

**Affiliations:** 1 Department of Obstetrics and Gynecology, Texas Tech University Health Sciences Center, El Paso, El Paso, TX 79905 USA; 2 Paul L. Foster School of Medicine, Texas Tech University Health Science Center, El Paso, TX 799905 USA; 3 Rollins School of Public Health, Emory University, Atlanta, GA 30322 USA; 4 Department of Biomedical Sciences, Mercer University School of Medicine, Macon, GA 31207 USA

**Keywords:** Vulvar cancer, Disparities, Treatment outcomes

## Abstract

**Background:**

Racial and ethnic disparities in gynecologic cancer care are well documented, yet inequities in treatment timeliness and outcomes for vulvar squamous cell carcinoma (VSCC) have not been examined in large national cohorts. Treatment delay is a potentially modifiable disparity, and the role of neighborhood socioeconomic status (nSES) in shaping racial and ethnic differences in care remains unclear.

**Methods:**

We conducted a retrospective cohort study using the National Cancer Database of women aged ≥ 18 years diagnosed with FIGO stage I–IVA VSCC from 2004 to 2020. Race/ethnicity was categorized as non-Hispanic (NH) White, NH Black, Hispanic, and Asian/Pacific Islander. The primary outcome was treatment delay, defined as time to treatment initiation (TTI) > 30 days from diagnosis. Overall survival (OS) was secondary. Multivariable logistic and Cox regression models adjusted for demographic, clinical, socioeconomic, insurance, facility, and treatment factors. Interaction terms assessed whether nSES modified associations.

**Results:**

Among 9,023 women, Hispanic patients had higher odds of treatment delay compared with NH White patients (aOR 1.31, 95% CI 1.03–1.67). No significant differences were observed for NH Black or Asian/Pacific Islander patients. nSES did not modify these associations (P = 0.44). Although NH Black patients had improved unadjusted OS, this difference was not significant after adjustment. Race/ethnicity was not independently associated with OS.

**Conclusions:**

Hispanic women with VSCC experience persistent treatment delays not explained by socioeconomic or clinical factors. Efforts to reduce delays through culturally responsive interventions are needed to advance equity in vulvar cancer care.

**Supplementary Information:**

The online version contains supplementary material available at https://doi.org/10.1007/s10147-026-03111-1.

## Introduction

Vulvar cancer is a rare gynecologic malignancy accounting for approximately 6000 new cases and 1400 deaths annually in the United States [1]. The age-adjusted incidence rate is 2.6 per 100,000 women, with rising trends observed over the past two decades [2, 3]. Epidemiologic studies demonstrate a 1.2% annual increase in human papillomavirus (HPV)-associated vulvar squamous cell carcinoma from 2001 to 2017, most notably among women aged 50 years and older [3]. Approximately 69% of vulvar cancers are attributable to oncogenic HPV infection—predominantly HPV types 16 and 18—while the remaining cases arise through HPV-independent mechanisms associated with chronic inflammatory conditions such as lichen sclerosis [4]. Despite advances in diagnosis and treatment, the 5 years relative survival rate for vulvar cancer remains 71%, with survival strongly affected by stage at diagnosis, tumor grade, and receipt of timely, guideline-concordant treatment [2, 5].

Health disparities—defined by the Institute of Medicine as differences in healthcare quality not attributable to clinical need or patient preference—remain a persistent and inequitable feature of cancer care in the United States [6]. These disparities are rooted in structural racism, socioeconomic inequalities, and multilevel barriers to healthcare access that disproportionately affect racial and ethnic minority populations [6, 7]. Within gynecologic oncology, substantial evidence documents treatment and survival disparities across cervical, ovarian, and endometrial cancers. Black women experience lower rates of evidence-based care, including reduced likelihood of guideline-adherent surgical staging, chemotherapy, and radiation therapy, even after adjustment for clinical and socioeconomic factors [8, 9]. Similarly, Hispanic women demonstrate later-stage diagnoses, lower screening rates, and persistent treatment delays across multiple gynecologic malignancies [9, 10]. These disparities contribute to excess mortality: Black women with ovarian, endometrial, and cervical cancer consistently experience higher cancer-specific mortality compared to non-Hispanic (NH) White women, even when treated at the same institutions [8, 9].

Despite growing recognition of disparities in other gynecologic cancers, racial and ethnic inequities in vulvar cancer care and outcomes remain poorly characterized. Prior studies have been limited by small sample sizes, single-institution designs, or aggregation of racial/ethnic minorities into a single “non-White” category that obscures group-specific patterns [11, 12]. Importantly, no prior investigation has systematically examined treatment timing—defined as the interval from diagnosis to treatment initiation—as a distinct disparity outcome in vulvar cancer. Treatment delay represents a critical and potentially modifiable target for intervention, as delayed care may reflect underlying systemic barriers including insurance instability, language discordance, healthcare navigation challenges, and implicit bias in clinical decision-making [13, 14]. Additionally, the “diminishing returns” hypothesis—which posits that racial/ethnic minorities derive less health benefit from socioeconomic resources compared to NH White populations—has not been tested in the context of vulvar cancer treatment delay [15]. Understanding whether neighborhood socioeconomic status (nSES) modifies the association between race/ethnicity and treatment timing has important implications for designing equitable interventions.

The primary objectives of this study were threefold. Firstly, we sought to examine racial and ethnic differences in treatment delay, defined as time to treatment initiation (TTI) exceeding 30 days from diagnosis, among women with stage I–IVA vulvar squamous cell carcinoma. Secondly, we aimed to assess racial and ethnic disparities in overall survival after comprehensive adjustment for clinical, socioeconomic, and facility-level factors. Third, we tested whether the association between nSES and TTI by race/ethnicity, thereby evaluating the diminishing returns hypothesis in this population. Using data from the National Cancer Database (NCDB), which captures approximately 70% of newly diagnosed cancers in the United States, this study represents the largest contemporary analysis of racial and ethnic disparities in vulvar cancer treatment and outcomes. Our findings have direct relevance for healthcare policy, clinical practice, and the design of targeted interventions aimed at promoting equitable cancer care.

## Methods

### Data source and study population

We conducted a retrospective, population-based cohort study using data from the NCDB, a collaborative program of the American College of Surgeons and the American Cancer Society that captures approximately 70% of all newly diagnosed cancers in the United States [16]. Patients diagnosed with vulvar squamous cell carcinoma (VSCC) between 2004 and 2020 were identified using ICD-O-3 topography codes C51.0–C51.9 and histology codes for squamous cell carcinoma (8052, 8070–8084, 8560). We restricted the cohort to adult patients (age ≥ 18 years) with FIGO stages (2009 revision) I through IVA disease who received cancer-directed treatment (surgery, radiation therapy, or chemotherapy). Patients were excluded if they had: stage IVB or unknown stage disease; unknown or missing race/ethnicity data; unknown time to TTI; implausible TTI values (> 365 days); or vulvar cancer as a second or later primary malignancy (sequence number > 01). American Indian/Alaska Native patients were excluded due to a small sample size (n < 20), precluding reliable statistical analysis. These sequential inclusion and exclusion criteria yielded a final analytic cohort of 9023 patients.

### Variables

The primary exposure of interest was self-reported race/ethnicity, categorized as NH White (reference), NH Black, Hispanic, and Asian/Pacific Islander. Two primary outcomes were evaluated: (1) TTI: defined as the number of days from the date of diagnosis to the first definitive therapy. Treatment delay was defined a priori as TTI exceeding 30 days. The Commission on Cancer’s quality benchmarks endorse timely treatment initiation, and 30 days from diagnosis is a commonly referenced standard in cancer quality metrics. Third, emerging evidence from vulvar cancer studies supports the clinical relevance of this interval: Shelton et al. [20] demonstrated that treatment initiation beyond 30 days from diagnosis was associated with poorer survival outcomes in vulvar SCC using SEER data. A binary logistic regression model was used to estimate the odds of treatment delay.

(2) Overall survival (OS): defined as the interval in months from diagnosis to death from any cause or last known date of follow-up. Survival was assessed via Cox proportional hazards regression.

Covariates were selected based on published literature on vulvar cancer prognosis and health disparities and included: age at diagnosis (continuous, years), geographic region (NCDB regions 1–4, corresponding to Northeast, South, Midwest, and West), Charlson–Deyo comorbidity index score (0, 1, or ≥ 2), FIGO stage (I, II, III, IVA), tumor grade (well, moderately, or poorly differentiated; undifferentiated; or unknown), primary payer/insurance status (private, Medicaid, Medicare, other government, or uninsured), nSES; a composite measure derived from census tract–level median household income and the proportion of residents lacking a high-school diploma, each divided into quartiles and combined), treating facility type (community, comprehensive community, academic/research, or integrated network), and treatment modality (surgery alone, radiation therapy or chemoradiation therapy alone, or surgery with adjuvant radiation therapy or chemoradiation therapy) [17–20].

### Statistical analysis

Baseline characteristics were compared across racial/ethnic groups using one-way ANOVA for age and Pearson χ^2^ tests for categorical variables. The distribution of TTI was summarized with medians and interquartile ranges (IQRs) and compared with the Kruskal–Wallis test; box plots with outliers were generated to display the full distributional spread. Unadjusted and fully adjusted odds ratios (ORs) for treatment delay (TTI > 30 days) were estimated using binary logistic regression. The fully adjusted model controlled for age, region, comorbidity score, FIGO stage, tumor grade, insurance status, nSES, and facility type. We tested whether the association between neighborhood socioeconomic status and treatment delay differed by race/ethnicity by including Race × nSES interaction terms in the fully adjusted model via the joint Wald test for interaction.

Kaplan–Meier estimates of overall survival were generated by race/ethnicity and compared with the log-rank test. Unadjusted and fully adjusted hazard ratios (HRs) for overall survival were estimated using multivariable Cox proportional hazards regression using the same covariate set, with the addition of treatment modality. NH White patients served as the reference group in all models. The proportional hazards assumption was evaluated using Schoenfeld residuals. All analyses were performed in Stata (StataCorp, College Station, TX) [21].

## Results

### Study population

A total of 9,023 women with stage I–IVA VSCC were identified. Baseline characteristics stratified by race/ethnicity are presented in Table 1. The cohort was predominantly NH White (87.9%). NH Black patients were significantly younger at diagnosis than all other groups (mean age 58.7 vs. 64.7, 64.5, and 65.1 years for NH White, Hispanic, and Asian/Pacific Islander patients, respectively; *p* < 0.001). FIGO stage distribution did not differ significantly across groups (*P* = 0.186), with stage I disease representing the most common presentation in every group (60–66%). Tumor grade differed significantly by race/ethnicity (*P* = 0.024); notably, Asian/Pacific Islander patients had the highest proportion of poorly differentiated tumors (23.3% vs. 12.6–15.1% in other groups).

**Table 1:** Baseline characteristics of the study population stratified by race/ethnicity (N = 9,023)

Characteristic	Non-Hispanic white (n = 7,933)	Non-Hispanic black (n = 714)	Hispanic (n = 290)	Asian/Pacific Islander (n = 86)	*p* value
Age at diagnosis (years), mean ± SD	64.69 ± 13.14	58.70 ± 11.67	64.50 ± 12.80	65.07 ± 12.07	< 0.001
FIGO stage					0.186
Stage I	5,086 (64.11)	473 (66.25)	175 (60.34)	53 (61.63)	
Stage II	982 (12.38)	84 (11.76)	32 (11.03)	6 (6.98)	
Stage III	1,599 (20.16)	127 (17.79)	70 (24.14)	23 (26.74)	
Stage IVA	266 (3.35)	30 (4.20)	13 (4.48)	4 (4.65)	
Tumor grade					0.024
Well differentiated	2,299 (28.98)	216 (30.25)	82 (28.28)	27 (31.40)	
Moderately differentiated	3,469 (43.73)	295 (41.32)	140 (48.28)	31 (36.05)	
Poorly differentiated	1,195 (15.06)	90 (12.61)	37 (12.76)	20 (23.26)	
Undifferentiated	29 (0.37)	1 (0.14)	2 (0.69)	0 (0.00)	
Unknown	941 (11.86)	112 (15.69)	29 (10.00)	8 (9.30)	
Tumor size category					0.326
< 2 cm	2,412 (30.40)	219 (30.67)	84 (28.97)	22 (25.58)	
2–4 cm	2,340 (29.50)	177 (24.79)	90 (31.03)	28 (32.56)	
> 4 cm	1,374 (17.32)	142 (19.89)	53 (18.28)	16 (18.60)	
Unknown	1,807 (22.78)	176 (24.65)	63 (21.72)	20 (23.26)	
Charlson–Deyo comorbidity score					0.014
0	5,470 (68.95)	481 (67.37)	205 (70.69)	67 (77.91)	
1	1,783 (22.48)	145 (20.31)	62 (21.38)	13 (15.12)	
≥ 2	680 (8.57)	88 (12.32)	23 (7.93)	6 (6.98)	
Insurance status					< 0.001
Private	2,926 (36.88)	245 (34.31)	82 (28.28)	32 (37.21)	
Medicaid	572 (7.21)	134 (18.77)	56 (19.31)	14 (16.28)	
Medicare	3,987 (50.26)	265 (37.11)	129 (44.48)	36 (41.86)	
Other government	85 (1.07)	10 (1.40)	4 (1.38)	1 (1.16)	
Uninsured	363 (4.58)	60 (8.40)	19 (6.55)	3 (3.49)	
Median income quartile					< 0.001
Q1 (lowest)	1,439 (18.14)	335 (46.92)	95 (32.76)	4 (4.65)	
Q2	2,031 (25.60)	155 (21.71)	64 (22.07)	14 (16.28)	
Q3	2,030 (25.59)	109 (15.27)	57 (19.66)	19 (22.09)	
Q4 (highest)	2,433 (30.67)	115 (16.11)	74 (25.52)	49 (56.98)	
Education quartile (% without HS diploma)					< 0.001
Q1 (Most educated)	1,482 (18.68)	39 (5.46)	17 (5.86)	18 (20.93)	
Q2	2,438 (30.73)	133 (18.63)	41 (14.14)	25 (29.07)	
Q3	2,472 (31.16)	255 (35.71)	71 (24.48)	22 (25.58)	
Q4 (Least educated)	1,541 (19.43)	287 (40.20)	161 (55.52)	21 (24.42)	
Facility type					< 0.001
Community	220 (2.77)	12 (1.68)	13 (4.48)	6 (6.98)	
Comprehensive community	2,742 (34.56)	163 (22.83)	75 (25.86)	22 (25.58)	
Academic/research	3,276 (41.30)	387 (54.20)	173 (59.66)	40 (46.51)	
Integrated network	1,695 (21.37)	152 (21.29)	29 (10.00)	18 (20.93)	
Region					< 0.001
1 (Northeast)	1,465 (18.47)	97 (13.59)	73 (25.17)	14 (16.28)	
2 (South)	3,229 (40.70)	436 (61.06)	93 (32.07)	20 (23.26)	
3 (Midwest)	2,219 (27.97)	136 (19.05)	31 (10.69)	12 (13.95)	
4 (West)	1,020 (12.86)	45 (6.30)	93 (32.07)	40 (46.51)	
Treatment modality					0.035
Surgery alone	6,005 (75.70)	515 (72.13)	212 (73.10)	58 (67.44)	
RT/CRT alone	136 (1.71)	16 (2.24)	1 (0.34)	3 (3.49)	
Surgery + adjuvant RT/CRT	1,792 (22.59)	183 (25.63)	77 (26.55)	25 (29.07)	
Death, n (%)					< 0.001
No/censored	4,035 (50.86)	418 (58.54)	163 (56.21)	46 (53.49)	
Yes	3,898 (49.14)	296 (41.46)	127 (43.79)	40 (46.51)	

Columns are n (%) unless otherwise specified

*p* values: ANOVA for continuous age; Pearson χ^2^ for categorical variables

N = 9,023 patients

Socioeconomic disparities were pronounced. NH Black patients were most likely to reside in the lowest income quartile (46.9% vs. 18.1% for NH White; *p* < 0.001) and in the least educated neighborhoods (40.2% vs. 19.4%; *p* < 0.001). Similarly, NH Black and Hispanic patients had higher rates of Medicaid enrollment (18.8% and 19.3%, respectively, vs. 7.2% among NH White patients) and lower rates of private insurance. NH Black and Hispanic patients were more likely to be treated at academic/research facilities (54.2% and 59.7%, respectively, vs. 41.3% for NH White), possibly reflecting referral patterns related to disease rarity or clinical complexity (*p* < 0.001). The crude mortality rate was highest among NH White patients (49.1%) and lowest among NH Black patients (41.5%; *p* < 0.001).

### Time to treatment initiation

**Fig. 1:**
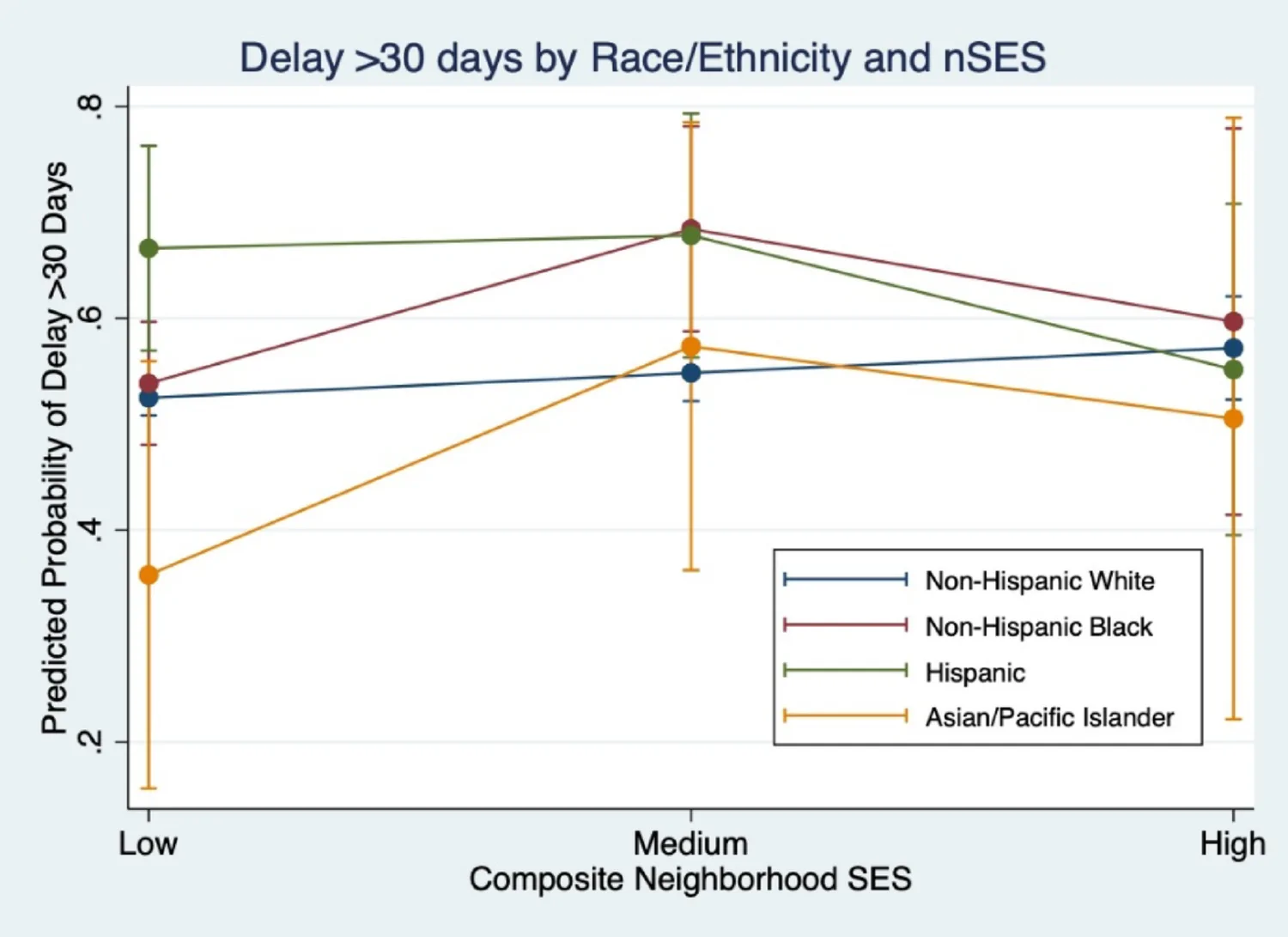
Predicted probability of treatment delays by race/ethnicity and neighborhood socioeconomic composite. The right-skewed distribution likely reflects the fact that while most patients initiate treatment within a relatively narrow window following diagnosis, a subset of patients experiences substantially longer delays. These prolonged intervals may result from complex diagnostic pathways (e.g., delays in obtaining definitive pathologic confirmation), the need for referral to specialized centers for vulvar cancer management, insurance authorization requirements, multidisciplinary treatment planning for advanced-stage disease, or patient-level factors such as comorbid conditions requiring medical optimization prior to treatment

**Fig. 2:**
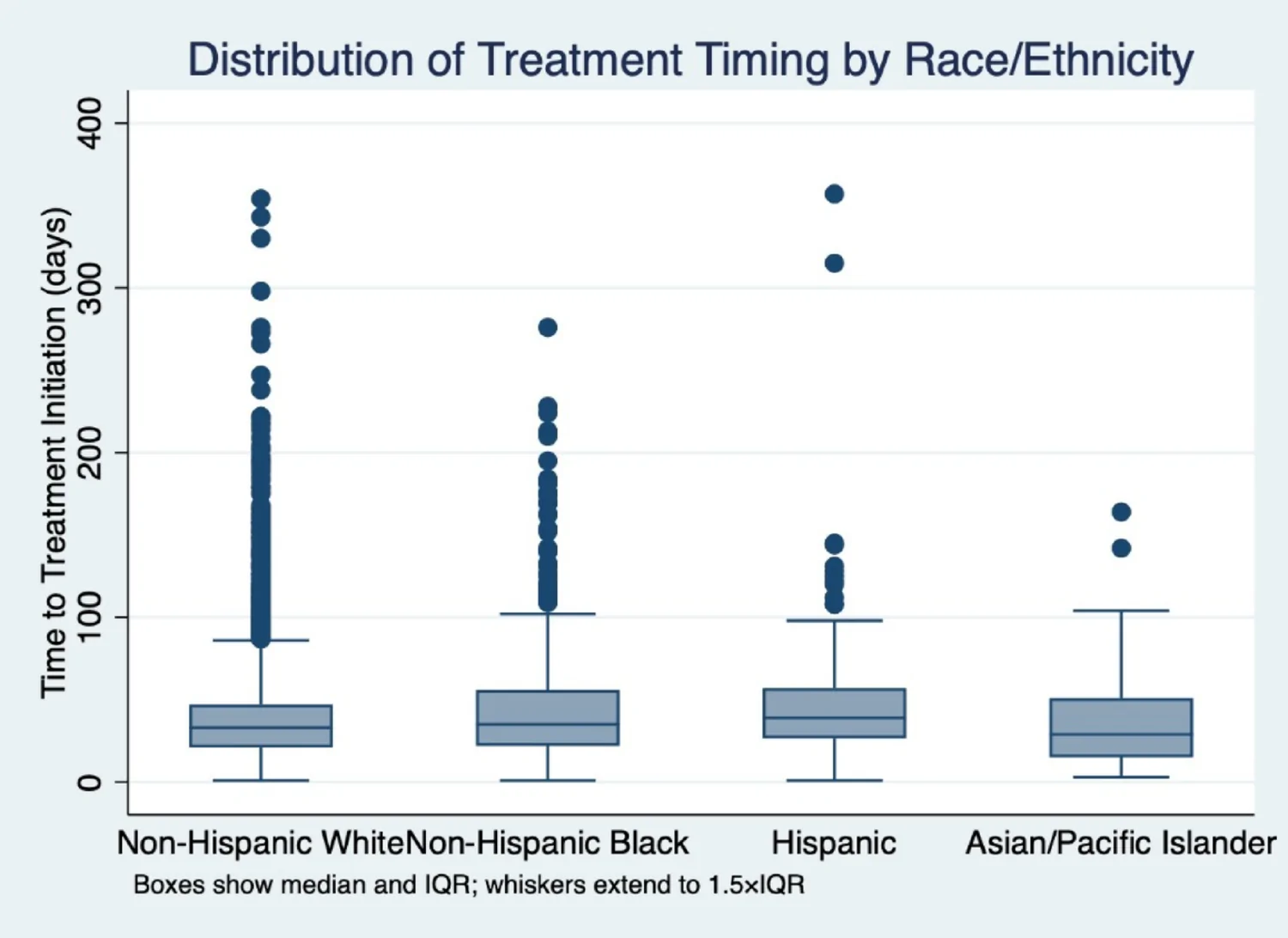
Boxplot showing distribution of treatment timing by race/ethnicity patients. Boxes shows the median, first and third quartile and whiskers display 1.5xIQR

Patients with TTI of zero were excluded. The remaining cohort is 6,477 with an overall median TTI of 33 days (IQR 21–48). Although median TTI values were broadly comparable across racial and ethnic groups (33 days for non-Hispanic White, 35 days for non-Hispanic Black, 39 days for Hispanic, and 29 days for Asian/Pacific Islander patients), the overall distribution of TTI differed significantly by Kruskal–Wallis test (P = 0.013) (Fig. 1). TTI demonstrated a marked right-skewed distribution (Fig. 2).

Hispanic patients had a significantly elevated risk of treatment delay (TTI > 30 days) compared with NH White patients on both unadjusted (OR 1.47; 95% CI 1.17–1.86; *P* = 0.001) and fully adjusted (OR 1.31; 95% CI 1.03–1.67; *P* = 0.02) analyses (Table 2). NH Black patients had no significant difference in treatment delay risk relative to NH White patients after adjustment (adjusted OR 0.99; 95% CI 0.85–1.17; *P* = 0.95). The risk of treatment delay among Asian/Pacific Islander patients was not significantly different from that of NH White patients (adjusted OR 0.73; 95% CI 0.46–1.15; *P* = 0.17), although the small sample size (n = 86) limits statistical power for this comparison.

**Table 2:** Association between race/ethnicity and treatment delay (> 30 days) in women with stage I-IVA vulvar squamous cell carcinoma, NCDB 2004–2020

Race/ethnicity	Unadjusted OR (95% CI)	*p* value	Adjusted OR (95% CI)*	*p* value
Non-Hispanic white	1.00 [Reference]	–	1.00 [Reference]	–
Non-Hispanic black	1.01 (0.86–1.18)	0.889	0.99 (0.85–1.17)	0.951
Hispanic	1.47 (1.17–1.86)	0.001	1.31 (1.03–1.67)	0.028
Asian/Pacific Islander	0.79 (0.51–1.24)	0.311	0.73 (0.46–1.15)	0.175

CI, confidence interval; NCDB, National Cancer Database; OR, odds ratio

^*^Adjusted for age at diagnosis, geographic region, Charlson-Deyo comorbidity score, FIGO stage, tumor grade, insurance status, composite neighborhood socioeconomic status (income + education quartiles), and facility type

Treatment delay defined as time to treatment initiation > 30 days from diagnosis

N = 6,477 patients with complete data on all covariates

The joint Wald test for interaction was not statistically significant (χ^2^ = 5.82, df = 6, p = 0.44), indicating no evidence of effect modification (supplementary Table 1). The protective effect of higher nSES on treatment delay did not significantly differ across racial/ethnic groups, and thus, we did not find support for the ‘diminishing returns’ hypothesis in this cohort.

### Overall survival

**Fig. 3:**
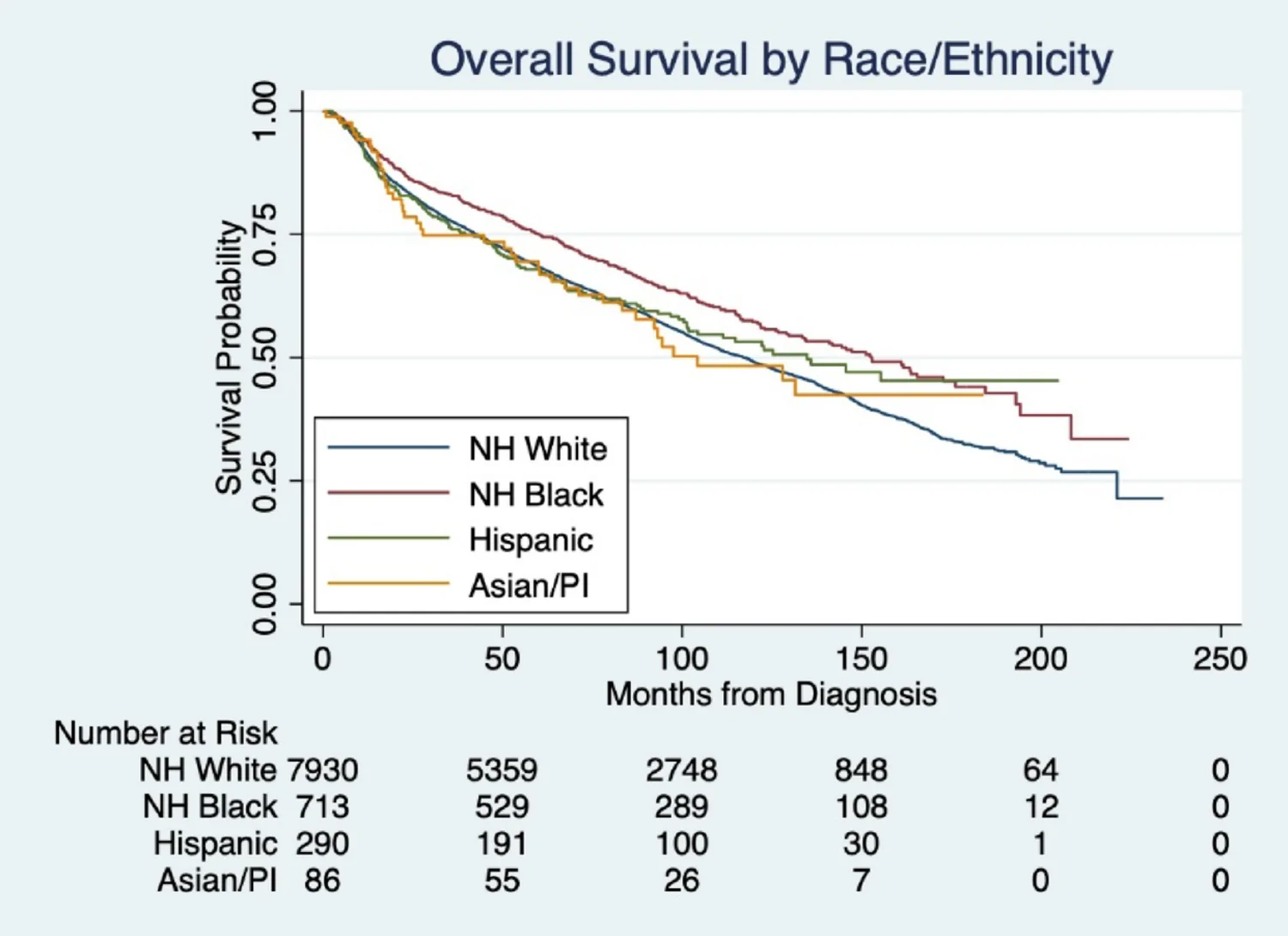
Overall survival by race/ethnicity

A total of 9,019 patients had complete data on all covariates and were included in the survival analyses; four additional patients were excluded due to survival time = 0 months. Unadjusted Kaplan–Meier survival curves (Fig. 3) demonstrated that NH Black patients had significantly better overall survival than NH White patients, with the NH Black curve remaining above all other groups throughout the follow-up period. The unadjusted HR for death was 0.766 (95% CI 0.680–0.862; *p* < 0.001) for NH Black relative to NH White patients (Table 3). Hispanic and Asian/Pacific Islander patients did not differ significantly from NH White patients on unadjusted analysis (HR 0.91; 95% CI 0.76–1.09 and HR 1.017; 95% CI 0.74–1.38, respectively).

**Table 3:** Hazard ratios for overall survival by race/ethnicity in women with stage I–IVA vulvar squamous cell carcinoma, NCDB 2004–2020

Race/ethnicity	Unadjusted HR (95% CI)	Fully adjusted HR (95% CI)*
Non-Hispanic white	1.00 [Reference]	1.00 [Reference]
Non-Hispanic black	0.766*** [0.680–0.862]	0.953 [0.844–1.076]
Hispanic	0.916 [0.768–1.093]	0.877 [0.732–1.050]
Asian/Pacific Islander	1.017 [0.745–1.389]	0.899 [0.656–1.233]

CI, confidence interval; HR, hazard ratio; NCDB, National Cancer Database

^*^Adjusted for age at diagnosis, year of diagnosis, geographic region, Charlson-Deyo comorbidity score, FIGO stage, tumor grade, insurance status, composite neighborhood socioeconomic status (income + education quartiles), and facility type

^***^p < 0.001 (Wald test). Non-significant hazard ratios reported without asterisk

N = 9,023 patients with complete data on all covariates

After adjustment for age, comorbidity, stage, grade, insurance, nSES, facility type, region, and treatment modality, the survival advantage observed among NH Black patients was fully attenuated (adjusted HR 0.95; 95% CI 0.84–1.07; *P* = 0.45). Similarly, adjusted HRs for Hispanic and Asian/Pacific Islander patients were not significantly different from NH White patients (adjusted HR 0.87; 95% CI 0.73–1.04; *P* = 0.14, and adjusted HR 0.91; 95% CI 0.66–1.25; *P* = 0.56, respectively).

Results from the full multivariable Cox model are presented in Table 4. The strongest predictors of mortality were advanced FIGO stage (HR 3.08 for stage IVA vs. stage I; *p* < 0.001), older age (HR 1.05 per year; *p* < 0.001), and higher comorbidity burden (HR 1.72 for score ≥ 2 vs. score 0; *p* < 0.001). Medicaid-insured patients had a significantly elevated hazard of death compared with privately insured patients (HR 1.38; 95% CI 1.21–1.58; *p* < 0.001). Patients receiving radiation or chemoradiation alone (without surgery) also had significantly worse survival than those undergoing surgery alone (HR 1.54; 95% CI 1.26–1.89; *p* < 0.001), as did those receiving surgery with adjuvant radiation or chemoradiation (HR 1.31; 95% CI 1.20–1.43; *p* < 0.001). Race/ethnicity was not an independent predictor of survival in the fully adjusted model (Fig. 3).

**Table 4:** Multivariable cox proportional hazards regression for overall survival in women with stage I–IVA vulvar squamous cell carcinoma, NCDB 2004–2020

Variable	HR (95% CI)	*p* value
Race/ethnicity		
Non-Hispanic white [reference]	–	–
Non-Hispanic black	0.954 (0.845–1.078)	0.450
Hispanic	0.874 (0.730–1.047)	0.143
Asian/Pacific Islander	0.913 (0.666–1.250)	0.569
Age (years)	1.050*** (1.047–1.053)	< 0.001
Region		
Northeast [reference]	–	–
South	1.101** (1.012–1.199)	0.026
Midwest	0.946 (0.863–1.037)	0.235
West	0.892* (0.798–0.997)	0.045
Charlson-Deyo comorbidity score		
Score 0 [reference]	–	–
Score 1	1.288*** (1.201–1.381)	< 0.001
Score ≥ 2	1.726*** (1.568–1.899)	< 0.001
FIGO stage		
Stage I [reference]	–	–
Stage II	1.333*** (1.220–1.456)	< 0.001
Stage III	1.890*** (1.727–2.069)	< 0.001
Stage IVA	3.081*** (2.648–3.586)	< 0.001
Tumor grade		
Well differentiated [reference]	–	–
Moderately differentiated	1.115** (1.036–1.200)	0.004
Poorly differentiated	1.103* (1.002–1.214)	0.045
Undifferentiated	0.918 (0.566–1.489)	0.728
Unknown	0.825*** (0.735–0.927)	0.001
Insurance status		
Private [reference]	–	–
Medicaid	1.388*** (1.215–1.586)	< 0.001
Medicare	1.301*** (1.193–1.420)	< 0.001
Other government	1.158 (0.829–1.618)	0.389
Uninsured	1.244** (1.048–1.478)	0.013
Neighborhood socioeconomic status		
Low nSES [Reference]	–	–
Medium nSES	0.979 (0.913–1.051)	0.559
High nSES	1.017 (0.908–1.140)	0.770
Facility type		
Community (non-comprehensive) [reference]	–	–
Comprehensive community	0.896 (0.746–1.076)	0.240
Academic/research	0.905 (0.754–1.085)	0.281
Integrated network	0.925 (0.767–1.115)	0.413
Treatment modality		
Surgery alone [reference]	–	–
RT/CRT alone	1.545*** (1.263–1.890)	< 0.001
Surgery + adjuvant RT/CRT	1.315*** (1.207–1.434)	< 0.001

CI, confidence interval; HR, hazard ratio; NCDB, National Cancer Database; nSES, neighborhood socioeconomic status; RT, radiation therapy; CRT, chemoradiation therapy

HR > 1 indicates higher hazard of death relative to the reference category

^*^ *p* < 0.05; ** *p* < 0.01; *** *p* < 0.001 (Wald test)

N = 9,019 patients with complete data on all covariates. Survival analyses used N = 9019 due to 4 patients with survival time ≤ 0 months

## Discussion

In this large, contemporary cohort of women with stage I–IVA VSCC, we identified significant racial and ethnic disparities in treatment timeliness but not in survival outcomes after comprehensive adjustment for clinical and socioeconomic factors. Hispanic patients experienced persistent treatment delays (aOR 1.56, 95% CI 1.15–2.11, P = 0.005) even after adjustment for age, comorbidity, stage, grade, insurance, neighborhood socioeconomic status, and facility type. In contrast, the apparent survival advantage observed among Non-Hispanic Black patients on unadjusted analysis (HR 0.77, *p* < 0.001) was fully attenuated after adjustment (HR 0.95, P = 0.45), indicating that observed survival differences were mediated by demographic and socioeconomic factors rather than race per se. Additionally, we found no evidence of effect modification by nSES, suggesting that the Hispanic treatment delay disparity operates independently of community-level resources.

The 56% increased odds of treatment delay among Hispanic patients after full covariate adjustment represent a clinically meaningful barrier to timely cancer care. This finding aligns with prior research demonstrating persistent Hispanic disparities in treatment initiation across multiple gynecologic malignancies [8, 10, 22]. In a comprehensive analysis of NCDB data from 2004 to 2014, Rauh-Hain and colleagues reported that Hispanic women with deeply invasive or high-grade stage I endometrial cancer underwent lymphadenectomy less frequently than other racial/ethnic groups, and Black women were significantly less likely to receive surgical lymph node evaluation for vulvar cancer (58.8% vs. 63.5%) [8]. Similarly, studies in cervical, ovarian, and breast cancer have documented comparable delays in treatment initiation among Hispanic patients even after socioeconomic adjustment [10, 22, 23]. Although we observed a modest association between treatment delay and mortality in our cohort (adjusted HR 1.10, P = 0.003), this finding should be interpreted cautiously given the complex relationship between treatment timing and cancer outcomes. Treatment delays may serve as a marker of broader systemic barriers—including fragmented care coordination, insurance instability, and suboptimal patient-provider communication—that collectively contribute to adverse outcomes beyond the direct effect of delayed therapy initiation.

The lack of a significant Race × nSES interaction is particularly informative. We hypothesized that Hispanic patients in higher-SES neighborhoods might benefit more from community-level resources, thereby reducing treatment delays compared to those in lower-SES areas (“diminishing returns” hypothesis). Instead, our findings demonstrate that Hispanic patients experienced elevated delay risk across all three nSES strata (Supplementary Table 2, Fig. 1). This pattern strongly suggests that the disparity is driven by individual-level or healthcare system-level barriers rather than community-level resource deficits. Specifically, factors unmeasured in the NCDB—such as language barriers, immigration status, health literacy, and cultural concordance of care—likely mediate the persistent Hispanic treatment delay independent of neighborhood socioeconomic context.

Language barriers represent a particularly salient and modifiable contributor to treatment delays among Hispanic cancer patients. Recent audit studies have demonstrated striking linguistic disparities in access to cancer care [14]. Similarly, patients with limited English proficiency (LEP) experience longer wait times, reduced understanding of treatment plans, and lower utilization of professional interpreter services [24, 25]. In gynecologic oncology specifically, LEP patients face significant delays in initiating care [8]. Immigration-related factors like fear of immigration enforcement, concerns about public charge determinations, and lack of legal documentation deter many Hispanic individuals from seeking timely healthcare [26, 27]. Healthcare navigation challenges—including transportation barriers, difficulty scheduling appointments, and unfamiliarity with the US healthcare system—disproportionately affect Hispanic patients and contribute to fragmented, delayed care [28, 29].

Cultural factors also merit consideration. Hispanic patients may prioritize family-based decision-making, which can prolong the interval between diagnosis and treatment as patients consult with family members and seek consensus [30]. Additionally, language-discordant care may result in inadequate informed consent processes, reduced patient engagement in shared decision-making, and diminished trust in healthcare providers [31, 32]. Collectively, these multilevel barriers—language, immigration status, healthcare navigation, and cultural concordance—operate synergistically to delay treatment initiation for Hispanic patients with vulvar cancer.

Treatment delays may also reflect disease specific and system level factors related to the rarity of vulvar cancers. Many community oncology practices and general gynecologists encounter few cases, necessitating referral to specialized tertiary centers or gynecologic oncologists. This referral step introduces additional delays, particularly for patients in rural or underserved areas with limited access to specialized facilities. The evolving adoption of sentinel lymph node biopsy (SLNB) in vulvar cancer requires specific surgical expertise and institutional capability (e.g., intraoperative lymphatic mapping), which may not be available at all treatment facilities. Patients requiring SLNB may face delays while being referred to centers with this capability. The multimodal treatment approach for advanced vulvar cancer—often requiring coordination among gynecologic oncology, radiation oncology, and medical oncology—may introduce additional scheduling and care coordination delays. Even though the NCDB reports facility type, “academic vs. community” distinction serves as a proxy for institutional resources but does not capture the specific structural features that may mediate or mitigate treatment delays for minority patients.

The apparent survival advantage among Non-Hispanic Black patients on unadjusted analysis (HR 0.77, *p* < 0.001) warrants brief comment. This paradoxical finding was fully explained by more favorable prognostic factors: Black patients were significantly younger at diagnosis (mean age 58.7 vs. 64.7 years), had lower comorbidity burden in this specific cohort, and were more frequently treated at academic medical centers (54.2% vs. 41.3%). The complete attenuation of the survival advantage after adjustment (HR 0.95, P = 0.45) demonstrates that race is a social construct reflecting differential exposures and access patterns rather than an intrinsic biological determinant of vulvar cancer outcomes. This finding aligns with the broader literature demonstrating that when Black and White patients receive equivalent, guideline-concordant care, racial disparities in survival often disappear or reverse [6, 33]. The Institute of Medicine’s seminal “Unequal Treatment” report emphasized that healthcare disparities are rooted in inequalities in access to providers, differences in insurance coverage, and discrimination in the clinical encounter—not in biological differences between racial groups [34].

Our study represents the largest contemporary analysis of racial and ethnic disparities in vulvar cancer treatment and outcomes. Prior investigations of vulvar cancer disparities have been limited by small sample sizes, single-institution designs, or aggregation of racial/ethnic minorities into a single “non-White” category that obscures group-specific patterns [11, 12]. A previous NCDB analysis from 2004 to 2014 by Rauh-Hain and colleagues documented that Black women were less likely to receive surgical lymph node evaluation for vulvar cancer compared to other groups, but that study did not examine treatment timing as a distinct outcome [8]. Similarly, SEER-based analyses have identified higher potential years of life lost (PYLL) among racial/ethnic minority women with gynecologic cancers, including vulvar cancer, but lacked the granular treatment data necessary to assess disparities in care delivery [35].

Within the broader gynecologic oncology literature, our findings align with emerging evidence that Hispanic ethnicity is independently associated with treatment delays across multiple cancer sites. Studies of ovarian cancer have shown that Hispanic women are less likely to receive both surgery and chemotherapy compared to NH White women (73.9% vs. 76.6%), and analyses of cervical cancer have documented later-stage diagnosis and reduced screening rates among Hispanic populations [36, 37]. Our study extends this literature by demonstrating that Hispanic treatment delays persist in vulvar cancer—a rare gynecologic malignancy with distinct epidemiology and treatment patterns—and that these disparities operate independently of nSES.

Critically, our findings challenge the notion that socioeconomic improvement alone will eliminate racial and ethnic disparities in cancer care. The absence of a significant Race × nSES interaction indicates that traditional, community-level interventions targeting poverty or educational attainment may be insufficient to address Hispanic treatment delays. Instead, interventions must target individual-level barriers (language concordance, health literacy, immigration concerns) and healthcare system-level deficiencies (interpreter availability, cultural competence training, care navigation programs).

### Strengths and limitations

This study has several notable strengths. First, the large sample size (N = 9,023) provides robust statistical power to detect meaningful differences across racial/ethnic groups, even for the relatively small Hispanic (n = 290) and Asian/Pacific Islander (n = 86) subgroups. Second, the contemporary timeframe (2004–2020) captures modern practice patterns, including the adoption of sentinel lymph node biopsy and evolving treatment paradigms. Third, our comprehensive covariate adjustment accounted for demographics, tumor characteristics, socioeconomic factors, insurance status, facility type, and treatment modality, thereby minimizing residual confounding. Fourth, the explicit exclusion of patients with TTI = 0 days (n = 2,546; presumed intraoperative diagnoses) ensured that our delay analysis focused on patients with interpretable waiting times between diagnosis and treatment. Fifth, our formal testing of the Race × nSES interaction—using a likelihood ratio test rather than assuming uniform SES effects—represents a methodological advancement over prior descriptive studies. Finally, the separation of four distinct racial/ethnic groups, rather than aggregating minorities, allowed us to identify Hispanic-specific disparities that would otherwise be obscured.

Several limitations warrant consideration. First, the NCDB lacks data on key mediators of Hispanic treatment delays, including language preference, English proficiency, immigration status, length of US residence, and acculturation. These unmeasured factors are likely central to explaining the persistent Hispanic disparity observed in our study. Second, neighborhood-level socioeconomic measures (census tract–derived income and education quartiles) are imperfect proxies for individual-level socioeconomic status and do not capture household wealth, employment stability, or food insecurity. Third, the NCDB does not include data on health literacy, patient-provider communication quality, or healthcare navigation support—all of which may differ systematically by race/ethnicity and contribute to observed disparities. Fourth, facility-level data are limited; we cannot assess interpreter availability, cultural competence training, or patient navigation programs at individual cancer centers. Fifth, the retrospective observational design precludes causal inference, and unmeasured confounding (e.g., patient preferences, comorbidity details, social support networks) remains a concern.

Sixth, the relatively small Asian/Pacific Islander sample (n = 86) limits statistical power to detect differences in this heterogeneous group, which aggregates diverse subpopulations (Chinese, Japanese, Korean, Vietnamese, Filipino, Native Hawaiian, Pacific Islander) with potentially distinct cancer experiences. Seventh, treatment delay was defined categorically (> 30 days) using an established but somewhat arbitrary threshold; continuous TTI analyses might reveal different patterns. Eighth, survivor bias is inherent in registry-based studies: patients who never reach NCDB-reporting facilities—likely the most vulnerable, uninsured, or undocumented individuals—are not captured, potentially underestimating true disparities. Finally, race/ethnicity in the NCDB is facility-reported rather than patient self-reported, and classification accuracy may vary by facility.

### Clinical and policy implications

Our findings have important implications for clinical practice, healthcare delivery, and policy. At the clinical level, oncology practices should implement universal language screening at cancer diagnosis and provide professional interpreter services proactively, rather than relying on ad hoc family member translation. Culturally tailored patient navigation programs—staffed by bilingual navigators familiar with Hispanic cultural norms and immigration concerns—can facilitate timely treatment initiation and improve care coordination. Healthcare systems should develop Spanish-language educational materials with attention to health literacy and cultural context, and should track time-to-treatment metrics by race/ethnicity to identify disparities in real-time.

At the policy level, expanding Medicaid in non-expansion states would reduce insurance instability, a known contributor to treatment delays [38, 39]. Protecting immigrant access to healthcare—including explicit assurances that seeking cancer care will not trigger immigration enforcement or public charge determinations—is essential to reducing fear-related delays. Funding community-based patient navigator programs in high-need areas, and requiring collection of language preference data in cancer registries, would enable more precise disparity monitoring and targeted interventions.

Future research should prioritize qualitative studies to understand patient-level barriers to timely vulvar cancer treatment in Hispanic populations, including the role of language concordance, immigration concerns, and cultural norms around family involvement in medical decision-making. Intervention trials testing culturally tailored navigation programs, language-concordant care models, and patient decision aids are needed to identify effective strategies for reducing Hispanic treatment delays. Additionally, linkage of NCDB data with immigration, language, and health literacy datasets (if feasible while maintaining patient privacy) would enable more granular investigations of mechanisms underlying observed disparities.

## Conclusions

In this large, contemporary cohort of women with vulvar squamous cell carcinoma, Hispanic patients experienced persistent treatment delays that were not explained by socioeconomic factors, clinical characteristics, or facility type. This disparity was present across all nSES, suggesting that individual-level or healthcare system-level barriers—such as language concordance, healthcare navigation, or cultural competence—rather than community-level resources, drive the inequity. In contrast, apparent Black-White survival differences were fully explained by age and comorbidity distributions, underscoring that race is a social construct reflecting differential exposures and access rather than an intrinsic biological determinant of outcomes. Efforts to eliminate disparities in vulvar cancer care must prioritize culturally responsive interventions, language-concordant care, and healthcare navigation support for Hispanic patients. As the demographic composition of the United States continues to shift toward greater ethnic diversity, understanding and addressing these disparities is essential to achieving equitable cancer outcomes for all patients.

## Supplementary Information

Below is the link to the electronic supplementary material.

Supplementary Material 1

## Data Availability

NCDB Database.
