# Supplementary material for: Racial disparities in time to treatment initiation and survival outcomes in patients with vulvar cancer: a national cancer database analysis
Source: Int J Clin Oncol. 2026 Jul 6;31(10):2102–13. doi: 10.1007/s10147-026-03111-1 (PMC13615111; doi:10.1007/s10147-026-03111-1)
Supplement: Supplementary file 1 [file 10147_2026_3111_MOESM1_ESM.docx]

| **Race/Ethnicity** | **N** | **Median TTI, days (IQR)** |
| --- | --- | --- |
| Non-Hispanic White | 5,731 | 33 (21–47) |
| Non-Hispanic Black | 475 | 35 (22–56) |
| Hispanic | 212 | 39 (26–57) |
| Asian/Pacific Islander | 59 | 29 (15–51) |
| **Overall** | **6,477** | **33 (21–48)** |

**Supplementary Table 1. Time to Treatment Initiation by Race/Ethnicity in Women with Stage I-IVA Vulvar Squamous Cell Carcinoma, NCDB 2004-2020**

Abbreviations: IQR, interquartile range (25th–75th percentile); NCDB, National Cancer Database; TTI, time to treatment initiation.

Kruskal–Wallis test: χ² = 25.105, *P* = 0.0001

**Supplementary Table 2. Test for Interaction Between Race/Ethnicity and Neighborhood Socioeconomic Status on Treatment Delay**

| **Outcome** | **Model** | **Interaction Tested** | **Test Statistic** | **Degrees of Freedom** | **P-value** |
| --- | --- | --- | --- | --- | --- |
| Treatment delay (categorical) | Multivariable regression | Race/Ethnicity × nSES | Likelihood ratio χ² = 4.12 | 6 | **0.66** |

Abbreviations: nSES, composite neighborhood socioeconomic status (income + education quartiles).

Treatment delay defined as time to treatment initiation >30 days from diagnosis. Multivariable model adjusted for age at diagnosis, year of diagnosis, geographic region, Charlson-Deyo comorbidity score, FIGO stage, tumor grade, insurance status, composite neighborhood socioeconomic status, and facility type.
